# Nouveau Formulaire Pratique des Hôpitaux

**Published:** 1833-10

**Authors:** 


*Nouveau Formulaire Pratique des Hôpitaux; ou, Choix de Formules des Hôpitaux, Civils et Militaires, de France, d'Angleterre, d'Allemagne, d'Italie, &c.: contenant l'Indication des Doses, auxquelles on administre les Substances Simples et les Préparations Magistrales et Officinales du Codex; l'emploi des Médicamens Nouveaux, et des Notions sur l'Art de Formuler.* Par MM. MILNE EDWARDS et P. VAVASSEUR, D.D. MM. Paris, 1832. Pp. 466.

THIS is a pocket volume containing an European Dispensatory, and certainly comprises a vast fund of information in a very small space. The medicines are not arranged according to the forms in which they are exhibited, as pills, powders, &c., but according to their therapeutic action. Thus, the first division consists of astringents, the next of tonics, and so on. This part of the work is preceded by some well-written observations on the art of prescribing, partly abridged from Gaubius. Thus, the authors dwell on the necessity of attending to idiosyncrasy, and give two remarkable instances of it; one from Gaubius, of a man in whom a small dose of powder of crab's-eyes produced all the symptoms of arsenical poisoning; and another, of a family attended by one of the compilers, in all the members of which castor-oil acts almost like a poison. They also mention the endermic method of administering remedies, remarking that it should be employed only when the medicine acts in very small doses, as morphia and strychnine.

The endermic method is very useful whenever we have reason to apprehend the irritating effect of a medicine on the mucous membrane lining the stomach and intestines, or when we fear that its qualities might be impaired by the digestive process.

Messrs. Edwards and Vavasseur give the well-known table of Gaubius for proportioning the doses of a remedy to the age of the patient: thus, if the quantity to be taken by an adult be represented by 1, a child two years old will take $\frac{1}{3}$, one of three years $\frac{1}{6}$, &c. This table is not very easy to recollect, and we therefore prefer the ingenious formula given by the late Dr. Young; a man, the profundity of whose genius could be equalled only by its versatility, and who was equally at home in the loftiest flights of physical science, and in the minutest details of practical art. Dr. Young says, "for children under twelve years old, the doses of most medicines must be diminished in the proportion of the age to the age increased by twelve: for example, at two years old, to $\frac{1}{7} = \frac{2}{2+12}$. At twenty-one, the full dose may be given." (*Med. Liter.* 2d edit., p. 453.) That is to say, the dose taken by an adult being called 1, the dose to be taken by a child will be represented by a fraction whose numerator is the age of the child, and the denominator is the age + 12.

Our authors observe (p. 11) that in general we ought to prefer simple to compound remedies; and, if we have recourse to the latter, we should still aim at simplicity as much as possible. We ought, say they, always to recollect the important maxim, "*Superflua nunquam non nocent.*" Surely the old maxim is "*Superflua non nocent*;" perhaps it has been improved by reversing it: but old saws should be quoted fairly.

"It is usual to put at the beginning of the first line of a prescription the sign ♃ or ℞, which is the abbreviation of the Latin word *recipe.*" (P. 17.) Gray, in his Supplement to the Pharmacopœia, gives a more ingenious, and perhaps a more correct explanation: he says, "R. Recipe, take: but for this the old authors, and the French to this day, use this sign ♃, being the old heathen invocation to Jupiter, seeking his blessing upon the formula, equivalent to the usual invocation of the poets, and of Mahomedan authors, or the *Laus Deo* with which bookkeepers and merchants' clerks formerly began their books of accounts and invoices; a practice not yet quite extinct." (4th edit. p. lv.)

At page 44 our authors give a receipt for "pilules styptiques," containing one fourth of a grain of acetate of lead in each pill, from two to four pills being a dose; and they add that, in the London hospitals, the dose of the acetate is carried to from one to three grains a-day; as if this were something remarkable. Now, in the preceding recipe, we are directed to make one drachm of acetate of lead into thirty-six pills, and the dose is to be from four to twelve a-day. As the French *gros,* represented by ʒi., contains seventy-two grains, each pill will contain two grains of the acetate of lead, and the patient will take from eight to twenty-four grains a-day. It is strange therefore that they should think the London dose a remarkable one.

We dwell on this subject, not so much for the purpose of pointing out the inconsistency of Drs. Edwards and Vavasseur, as to attract attention to the doses in which it is necessary to give the acetate of lead in urgent cases. Mr. Laidlaw, a very industrious practitioner, published an essay, about five years since, detailing several cases of menorrhagia cured by large doses, such as ten grains a-day, or more. Dr. Christison also says that he has often given it, in divided doses, to the amount of eighteen grains daily, for eight or ten days, without remarking any unpleasant symptom whatever, except once or twice slight colic; and he observes, that Mr. Daniell gave it in larger doses; and "Van Swieten even mentions a case in which it was given to the amount of a drachm daily for ten days before it caused any material symptom." *(Christison on Poisons,* p. 413.)

Under the head of *Excitans Généraux,* we find an anti-gonorrhœal draught, composed of two drachms of cubebs, two ounces of wine, and one drop of essence of bergamot. This is used at the Montpelier hospitals, and seems to us a very elegant formula. Of course the wine is of the lightest kind. There is also an enema, containing six drachms of cubebs, to be used in similar cases.

Under the head of *Opium,* we are informed (page 312) that ammonia, the carbonates of soda and of potass, corrosive sublimate, nitrate of silver, acetate of lead, the salts of copper, iron, and zinc, and the infusion of nutgalls, are incompatible with it; yet we have previously been directed to combine opium and acetate of lead in pills. We suspect that the combination is an exceedingly good one, practically, even though the opium may be partially decomposed.

At page 313 it is stated that eleven grains of Dover's powder contain one of opium. This is the case in the Parisian codex; in our Pharmacopœia the proportion is one in ten.

At the Montpelier hospitals things seem to be carried on in a truly regal style; for they have a sirop d'or and a pommade d'or, composed of gold dust, with gum and lard respectively. They are chiefly used in venereal cases.

This little manual is certainly, upon the whole, an excellent one, and does great credit to the tact of MM. Edwards and Vavasseur; and we think that, if it were translated by an able hand,—not merely done into English, but altered and anglicised, it would stand a good chance of becoming a popular work in this country.

